# Machine Learning Computing Migration and Management Based on Edge Computing of Multiple Data Sources in the Internet of Things

**DOI:** 10.1155/2022/8065767

**Published:** 2022-09-12

**Authors:** Yudong Yin

**Affiliations:** Xi'an Jiaotong University, Xi'an 710049, Shaanxi, China

## Abstract

With the implementation of the concepts of smart city and smart home, the number of user-intelligent terminal devices is increasing. The traditional computing framework cannot meet the increasing data volume and computing needs. Edge computing based on multiple data sources of the Internet of things can not only meet the computing needs of users' intelligent devices but also reduce energy consumption and user computing waiting time. Therefore, this article puts forward the research on the migration and management of deep reinforcement learning computing based on the edge computing of Internet of things multiple data sources, integrates the deep reinforcement computing technology in the edge computing of Internet of things multiple data sources, and optimizes the edge computing migration scheme and resource allocation management. The test results show that deep reinforcement learning can effectively control the cost of computing migration and enable it to complete computing tasks efficiently while maintaining stable operation. Compared with the traditional enhanced algorithm and the minimum migration scheme, the management model can complete the computing migration task with less energy consumption and shorter average computing waiting time.

## 1. Introduction

The Internet of things is an information carrier based on the Internet and telecommunication network. It is a network extended from the Internet. It realizes the connection between the intelligent terminal equipment and the Internet through a cellular network and realizes the purpose of connecting different independent ordinary individuals [1]. 5G technology provides communication technology support with low power consumption, low cost, low delay, and high rate for the development of the Internet of things and promotes the development of the concepts of the Internet of things, smart city, and smart furniture. The data volume generated by the increasing scale of intelligent terminal equipment increases exponentially, making the traditional computing framework unable to support the increasing data volume [2]. At the same time, the battery life of intelligent terminal equipment and its limited computing capacity cannot meet the needs of users, so it is necessary to carry out quantity calculation through the centralized cloud computing data center [3]. This undoubtedly improves the requirements for energy consumption, transmission bandwidth, and processing delay of cloud computing data center. If we want to ensure the real-time transmission of Internet of things data in the cloud, it cannot be realized only by cloud computing. Therefore, the concept of edge computing came into being. The difference between edge computing and cloud computing is that it is a decentralized computing architecture, that is, the intensive computing generated in intelligent terminal devices is migrated from the network central node to the network logical edge node so as to shorten the transfer process of data in the network and provide network services for faster data processing and computing [4]. Edge computing is realized through low-cost edge servers. Its information processing and storage capabilities not only greatly reduce the workload and pressure of cloud center servers but also meet the needs of intelligent terminal device users for real-time network services.

## 2. Related Works

Closely related to the concept of edge computing is the concept of computing migration and resource scheduling management. At present, there have been many studies on energy consumption and delay in the migration and resource scheduling management of edge computing. Some studies have used tree-based distributed hierarchical mechanism to set up network edge cloud servers close to users and proposed workload service placement algorithm based on the peak load analysis results of clouds at different levels [5]. Some literature have described the development process of edge computing and given its differences and relations with fog computing, sea cloud computing, and mobile edge computing [6]. Some literature pointed out that the important motivation of virtual machine migration in edge computing technology is user mobility and the real-time migration of virtual machine has a very important impact on the improvement of resource utilization and physical server efficiency. Therefore, many scholars have studied the real-time migration of virtual machine [7]. Computing migration is to migrate part or all of the application programs with intensive computing in the intelligent terminal device to the edge cloud so as to obtain rich computing and storage resources, assist the intelligent terminal device to complete the calculation of the corresponding application program in a short time through the strong processing capacity of the edge cloud, and achieve the purpose of reducing application execution time and saving equipment energy [8, 9]. In the full migration mode, some literature compare the time for the edge end and local execution to complete the computing task so as to determine whether the computing migration needs to be carried out [10]. Based on the energy consumption index in the working process, some literature give the evaluation criteria of calculation migration. If the energy consumed by calculation migration is greater than that consumed by local execution, local execution task is selected; otherwise, computing migration is carried out [11]. In addition, some studies divide the application degree according to fine granularity and arbitrarily divide it to the edge cloud and local for execution so as to speed up the speed of task execution [12]. In addition, research is based on multiuser mobile edge computing to migrate all or part of each user's tasks to the edge cloud, which can provide users with parallel computing services so as to shorten the task execution time [13, 14]. Most of the models considered in many studies are to select the single edge node closest to the intelligent terminal device for edge calculation when calculating all or part of the migration. In this case, the energy consumption and delay of the model algorithm are studied. Although this can optimize the use of edge nodes to the greatest extent, the factors such as uneven distribution of edge nodes and performance differences between nodes are not considered. Therefore, the optimization of edge node performance is of great value to the research of node selection in edge computing migration and resource scheduling management.

This article proposes the research on machine learning computing migration and management based on the edge computing of multiple data sources in the Internet of things. Deep reinforcement learning technology is introduced into the edge computing method of the Internet of things to optimize the problems such as high computing cost, long computing time, and resource scheduling.

## 3. Based on Edge Computing of Multiple Data Sources in the Internet of Things

**Figure 1:**
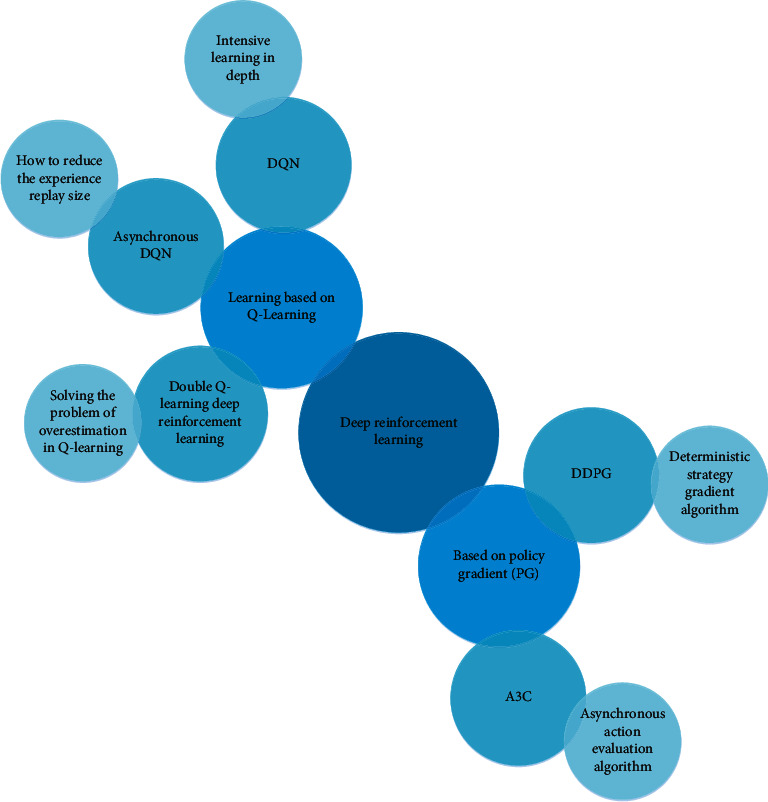
Classification diagram of the deep reinforcement learning algorithm.

Unlike supervised machine learning, in reinforcement learning, researchers train models by having an agent interact with the environment. When the agent's behavior produces the desired results, it gets positive feedback. It reflects human learning by exploring and receiving feedback from the environment. Deep learning has strong perception ability but it lacks certain decision-making abilities, and reinforcement learning has the ability of decision-making and is helpless to perceive problems. Therefore, the combination of the two provides a solution to the perception decision-making problem of complex systems. A deep reinforcement learning algorithm framework is a clever algorithm for decision learning that combines deep learning and reinforcement learning. It can complete the fitting of functions and models in reinforcement learning through the nonlinearity of deep learning so as to achieve the purpose of optimal decision-making. It has the advantages of deep learning and reinforcement learning [15].  Figure 1 is a classification diagram of the deep reinforcement learning algorithm.

In reinforcement learning, the value function-based reinforcement learning method selects the most valuable behavior in discrete actions according to the final reward and punishment value of different behaviors, while the probability-based strategy gradient reinforcement learning method has two advantages over the value function-based algorithm. On the one hand, when the algorithm based on the value function completes the action selection task in the continuous interval, the action is usually required to be limited and discrete, but the final effect is not ideal because all actions cannot be covered in the sampling process, or more complex special networks are designed due to optimization problems, making it difficult to solve the problem. The probability-based strategy gradient algorithm can arbitrarily select the actions in the continuous interval when performing the same task and directly optimize the objective function by adjusting the strategy parameters. On the other hand, the algorithm based on value function belongs to the greedy algorithm, in which the selection probability of the maximum action of the unique value function is close to 1, thus ignoring the random optimal strategy based on probability distribution. However, the strategy gradient algorithm based on probability can represent random strategies and can show the probability distribution of multiple actions when the strategy is in an uncertain state. Therefore, this article uses the DDPG algorithm in the policy gradient algorithm in deep reinforcement learning to optimize multiuser partial migration. The value function of traditional reinforcement learning is adopted in deep reinforcement learning. At the same time, reinforcement learning needs to obtain an action strategy in a specific state. Therefore, each strategy in DRL is divided into each execution action. The obtained action function is expressed as follows:

(1) $$\begin{array}{c} V^{\pi }\left(s\right)=\pi \left(s\right)\sum_{s^{\prime }\in s}p\left(s,s^{\prime }\right)\left[r_{0}+\gamma V^{\pi }\left(s^{\prime }\right)\right]\text{,} \end{array}$$

where the result is expressed as follows:

(2) $$\begin{array}{c} Q\left(s,a\right)=r_{s}^{a}+\gamma \sum_{s^{\prime }\in s}p\left(s,s^{\prime }\right)\sum_{a^{\prime }\in A}\pi \left(\left.a\right|s^{\prime }\right)Q\left(s^{\prime },a^{\prime }\right) \end{array}$$

The strategy is parameterized and assigned to the parameter vector *θ*, that is, *π*(*a*|*s*, *θ*), and the sampling trajectory *τ* is introduced, whose probability is expressed as *p*(*τ*|*θ*). The optimization goal is to maximize *R*(*τ*), and its expected value is shown as follows:

(3) $$\begin{array}{c} E_{\tau }\left[R\left(\tau \right)\right]=\sum_{\tau }R\left(\tau \right)p\left(\tau \left|\theta \right.\right)\text{,} \end{array}$$

where *R*(*τ*) after the derivation of *θ* is shown as follows:

(4) $$\begin{array}{c} \nabla_{\theta }E_{\tau }\left[R\left(\tau \right)\right]=E_{\tau }\left[R\left(\tau \right)\nabla_{\theta }\log\;p\left(\tau \left|\theta \right.\right)\right]\text{.} \end{array}$$

The probability chain method is used for formula (4) and the sum obtained after taking logarithm is derived with respect to *θ* to obtain formula (5):

(5) $$\begin{array}{c} \nabla_{\theta }E_{\tau }\left[R\left(\tau \right)\right]=E_{\tau }\left[R\left(\tau \right)\nabla_{\theta }\sum_{t=0}^{T-1}\log\;\pi \left(a_{t}\left|s_{t},\right.\theta \right)\right] \end{array}$$

where *τ*=(*s*_0_, *a*_0_, *s*_1_, *a*_1_, ..., *s*_*T*−1_, *a*_*T*−1_, *s*_*T*_).

In the general strategy gradient algorithm, there are always unselected actions that may correspond to greater benefits, and the probability of these actions in training will be lower. At the same time, the general strategy gradient algorithm belongs to the round update algorithm. If the benefit of the whole track is low, even if there are good actions, the probability of being selected in the next round will be reduced. To solve these problems, the overall benefit baseline of each round is set as *b*, and the benefit of each round may be negative after removing the benefit baseline. The probability of selection changes from slow increase to decrease so as to avoid the unselected action falling into a vicious circle, which is expressed as follows:

(6) $$\begin{array}{c} \nabla_{\theta }E_{\tau }\left[R\left(\tau \right)\right]\approx \frac{1}{N}\sum_{n=1}^{N}\sum_{t=0}^{T-1}R\left(\tau^{n}\right)\nabla_{\theta }\log\;\pi \left(a_{t}^{n}|s_{t}^{n},\theta \right)\\ \approx \frac{1}{N}\sum_{n=1}^{N}\sum_{t=0}^{T-1}\left[R\left(\tau^{n}\right)-b\right]\nabla_{\theta }\log\;\pi \left(a_{t}^{n}|s_{t}^{n},\theta \right) \end{array}$$

where *b* ≈ *E*_*τ*_[*R*(*τ*)]; the actual income corresponding to each specific action is adjusted to the cumulative income ∑_*t*′=*t*_^*T*−1^*r*_*t*′_^*n*^ − *b* of the action in the future, and the discount factor *γ* of the actual income is introduced to optimize *R*(*τ*^*n*^) into a function *A*^*θ*^(*a*_*t*_, *s*_*t*_) related to the current strategy, the state, and action of a specific step, which is expressed as follows:

(7) $$\begin{array}{c} \approx \frac{1}{N}\overset{N}{\underset{n=1}{\sum }}\overset{T-1}{\underset{t=0}{\sum }}A^{\theta }\left(a_{t},s_{t}\right)\nabla_{\theta }\log\;\pi \left(a_{t}^{n}|s_{t}^{n},\theta \right),b\approx E_{\tau }\left[R\left(\tau \right)\right]\text{.} \end{array}$$

As *a*_*t*_, *s*_*t*_ is random, *A*^*θ*^(*a*_*t*_, *s*_*t*_) is also a random variable with instability. On this basis, reinforcement learning algorithm based on value function and strategy gradient needs to be added to form a new algorithm.

**Figure 2:**
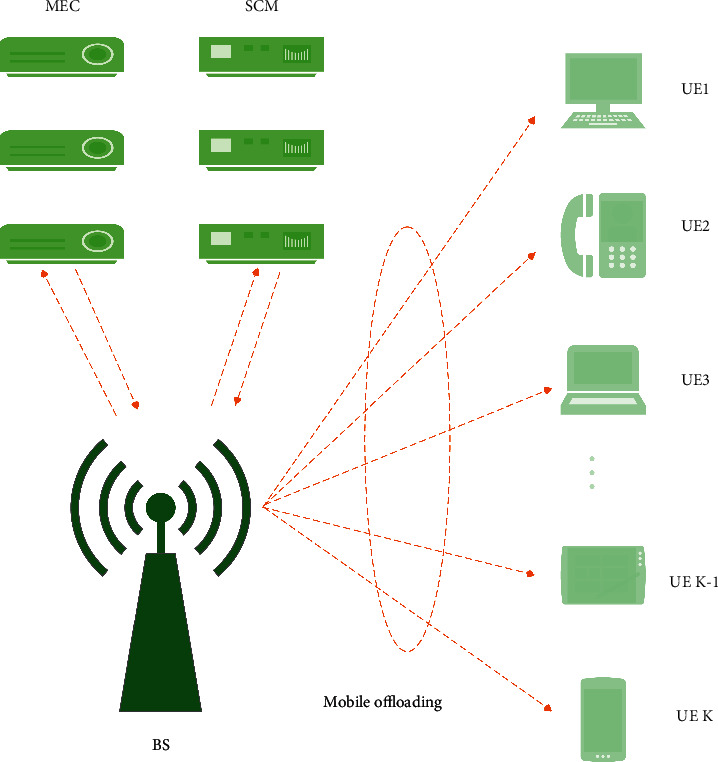
Specific system architecture diagram of single-cell multiuser complete migration.

In this article, the micro-cell manager is used to manage and schedule the cooperation between all user equipment and the base station for migration computing tasks. In the single-cell multiuser scenario, the connection between UE and micro-cell manager is completed through the base station and mobile edge computing server, and the communication mode of base station is TDMA. Let the tolerable delay constraint of users who choose to migrate to the base station be *L*_max_, which is also the duration of time slot. As shown in Figure 2, it is the system framework diagram.

Let the user equipment set connected to the base station be *k*={1,2, ..., *k*, ..., *K*}, and the rate at which the user equipment uploads data to the base station can be obtained according to the Nongxiang formula, which is expressed as follows:

(8) $$\begin{array}{c} R_{k}=B\;\log_{2}\;\left(1+P_{t,k}h_{k}^{2}/N_{0}\right)\text{,} \end{array}$$

where the broadband of complex Gaussian white noise channel is expressed as *B*, its variance is expressed as *N*_0_, the transmission power of user equipment is expressed as *P*_*t*,*k*_, and its channel gain is expressed as *h*_*k*_. If the application in the single-cell multiuser scenario is divided into subsets of various sizes and can flexibly select partial or complete migration, the decision vector composed of the migration proportion calculated by the user equipment is expressed as Λ=[*λ*_1_, *λ*_2_, ..., *λ*_*k*_, ..., *λ*_*K*_]. Assuming that the local computing capacity of each user equipment is expressed as *F*_*k*_(bits/s) and the local energy consumption of a cycle is expressed as *P*_*k*_, then the conditions that each user equipment needs to meet are expressed as follows

(9) $$\begin{array}{c} \left(1-\lambda_{k}\right)I_{k}/F_{k}\leq L_{\max}\text{.} \end{array}$$

The range of the respecified migration ratio is *λ*_*k*_(max {0, (1 − *L*_max_*F*_*k*_/*I*_*k*_)} ≤ *λ*_*k*_ ≤ 1), and the locally calculated energy consumption of user equipment is shown as follows:

(10) $$\begin{array}{c} E_{l,k}=\left(1-\lambda_{k}\right)I_{k}C_{k}P_{k}\text{.} \end{array}$$

The number of bits of input data calculated by the user equipment is expressed as *I*_*k*_, and the number of cycles calculated by each bit of data by the user equipment is expressed as *C*_*k*_.

Suppose that the allocation time for each user equipment to migrate data to the edge server is *t*_*k*_(*t*_*k*_ ≥ 0) and its decision vector is expressed as *t*_*k*_(*t*_*k*_ ≥ 0), the conditions for meeting the migration time of all user equipment are expressed as follows:

(11) $$\begin{array}{c} \sum_{k=1}^{K}t_{k}\leq L_{\max} \end{array}$$

*N*_0_(2^*R*_*k*_/*B*^ − 1)/*h*_*k*_^2^=*P*_*t*,*k*_ is obtained according to Shannon formula and transfer term, and the construction function is expressed as follows:

(12) $$\begin{array}{c} f\left(x\right)=\frac{N_{0}\left(2^{R_{k}/B}-1\right)}{h_{k}^{2}} \end{array}$$

where *R*_*k*_=*λ*_*k*_*I*_*k*_/*t*_*k*_; the energy consumption calculated by user equipment migration is expressed as follows:

(13) $$\begin{array}{c} E_{o,k}=P_{t,k}t_{k}=f\left(R_{k}\right)t_{k}=f\left(\lambda_{k}I_{k}/t_{k}\right)t_{k} \end{array}$$

When the local and migration are parallel, the calculation energy consumption is expressed as follows:

(14) $$\begin{array}{c} E_{k}\left(\lambda_{k},t_{k}\right)=E_{l,k}+E_{o,k}=\left(1-\lambda_{k}\right)I_{k}C_{k}P_{k}+f\left(\lambda_{k}I_{k}/t_{k}\right)t_{k}\text{.} \end{array}$$

Then the resource scheduling management problem based on the edge calculation of multiple data sources in the Internet of things is transformed into an optimization problem to minimize the total energy consumption by changing the decision vectors Λ=[*λ*_1_, *λ*_2_, ..., *λ*_*k*_, ..., *λ*_*K*_] and *T*=[*t*_1_, *t*_2_, ..., *t*_*K*_], which is shown as follows:

(15) $$\begin{array}{c} \left.\min_{\lambda_{k},t_{k}}\sum_{k=1}^{K}\left[\left(1-\lambda_{k}\right)I_{k}C_{k}P_{k}+f\left(\frac{\lambda_{k}I_{k}}{t_{k}}\right)\right.t_{k}\right]\begin{array}{c} s.t.C1:\sum_{k=1}^{K}\lambda_{k}I_{k}C_{k}\leq F_{c}\text{,}\\ C2:\max\;\left\{0,\left(1-\frac{L_{\max}F_{k}}{I_{k}}\right)\right\}\leq \lambda_{k}\leq 1\text{,}\\ C3:\sum_{k=1}^{K}t_{k}\leq L_{\max}\text{.} \end{array} \end{array}$$

*C*1, *C*2, *C*3 in the above formula represents the constraint condition, where *C*1 represents that the upper limit of the computing power of the edge computing server is the constraint condition of the decision vector. The edge calculator is mainly used by the edge server for data processing, so it will greatly improve the efficiency of data processing, shorten the time of data processing, and maintain the originality of data to a certain extent. Otherwise, when the migrated data exceed the bearing limit of the server, the corresponding calculation will not be completed within the time delay that users can tolerate. *C*2 means that when computing migration and local computing are parallel, the decision vector needs to be based on the local computing power of the user equipment, so that the parallel computing delay is within the delay constraint that the group compensation users can tolerate. *C*3 means that the sum of decision vectors can meet the delay range that all users can tolerate.

Therefore, the total energy consumption cost obtained after each scheduling is completed is expressed as follows:

(16) $$\begin{array}{c} \left.\left.E_{k}\left(\lambda_{k}\right.,t_{k}=\sum_{k=1}^{K}\left[\left(1-\lambda_{k}\right)I_{k}C_{k}P_{k}+\frac{t_{k}f\left(\lambda_{k}I_{k}/t_{k}\right)}{h_{k}^{2}}\right.\right)\right]\text{.} \end{array}$$

The edge computing capacity required by the scheduling scheme in practice is shown as follows:

(17) $$\begin{array}{c} P_{n}=\sum_{k=1}^{K}\lambda_{k}I_{k}C_{k}\text{.} \end{array}$$

Although a large number of edge servers deployed at the edge of the network bring convenience to users, the uncertainty of the number of mobile terminals is likely to lead to uneven load of edge servers and generate more energy consumption. This makes resource allocation in mobile edge computing face complex problems of energy consumption and utility optimization. The above total energy consumption overhead and the required computing power of the edge server are taken as the state after each execution action, that is, *s*(*s*_1_, *s*_2_). The state *s*_1_ can judge whether each scheduling scheme can achieve the purpose of minimizing the system energy consumption overhead, and the state *s*_2_ can judge whether the computing power of the edge server has been fully utilized.

The DDPG algorithm in the deep reinforcement learning algorithm, namely deep deterministic policy gradient, is a new algorithm based on actor critical algorithm and introduced dqn. In the actor critical algorithm, the depth enhancement algorithm adopted by the critical network is an algorithm based on the value function, which can output the corresponding *Q*^*π*^(*a*_*t*_^*n*^, *s*_*t*_^*n*^) and *V*^*π*^(*s*_*t*_^*n*^) according to the state and action, and the output can represent the random variable *A*^*θ*^(*a*_*t*_, *s*_*t*_), that is, *Q*^*π*^(*a*_*t*_^*n*^, *s*_*t*_^*n*^) − *V*^*π*^(*s*_*t*_^*n*^). Because the above network structure is not convenient, as shown in formula (17),

(18) $$\begin{array}{c} Q^{\pi }\left(a_{t}^{n},s_{t}^{n}\right)=E\left[r_{t}^{n}+V^{\pi }\left(s_{t+1}^{n}\right)\right] \end{array}$$

**Figure 3:**
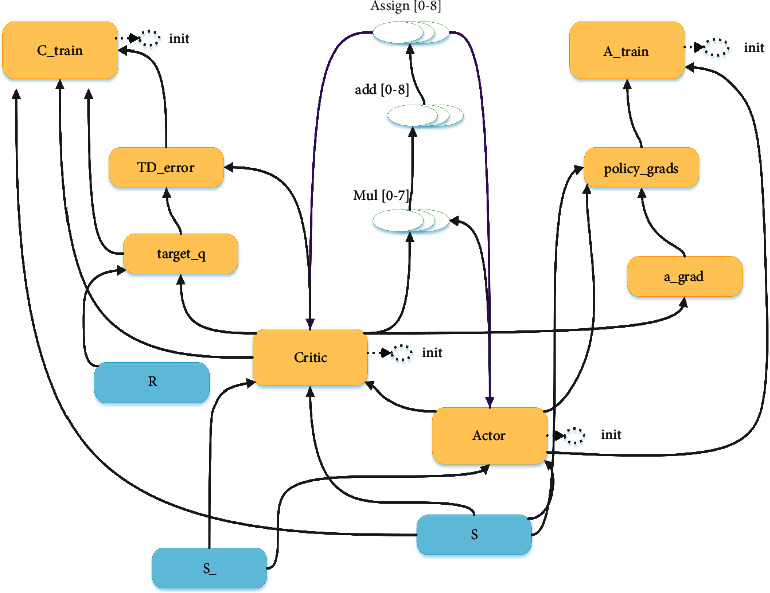
Algorithm specific network distribution diagram.

The approximate representation of random variables is *r*_*t*_^*n*^+*V*^*π*^(*s*_*t*+1_^*n*^) − *V*^*π*^(*s*_*t*_^*n*^), and *A*^*θ*^(*a*_*t*_, *s*_*t*_) is the weight function of critical network. The parameters of the two neural networks involved in the algorithm are updated in a continuous state every time, and there is a correlation between the parameters before and after the update so that the neural network cannot look at the problem in depth, and the training effect is not ideal. In the DDPG algorithm formed after the introduction of dqn, two mechanisms are used to update the parameters of different neural networks, which avoids the time correlation of parameters and improves the update efficiency. Random uniform sampling a small batch of data samples composed of training data samples for calculation. And in the process of gradient descent, the learning rate can decay itself, so it can reduce the variance of parameter update, and the convergence is more stable. At the same time, the algorithm can maximize the cumulative income of the objective function through the gradient rise update strategy.  Figure 3 shows the specific network distribution diagram of the algorithm.

## 4. Simulation Analysis of Reinforcement Learning Computing Migration Model

**Figure 4:**
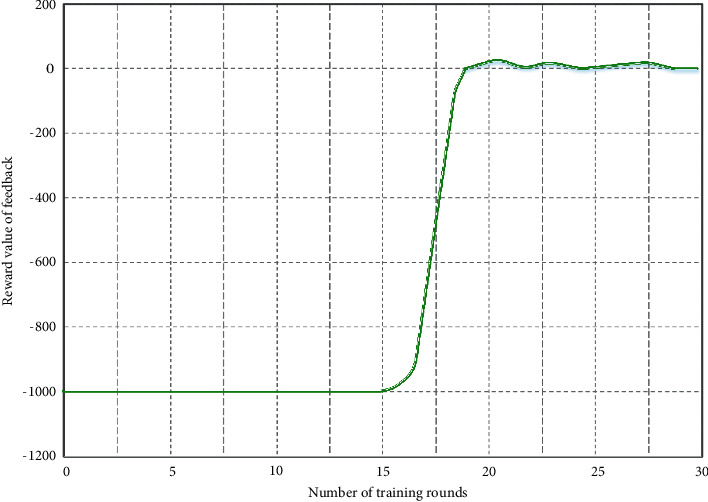
Convergence of deep reinforcement learning computing migration algorithm based on edge computing of multiple data sources in the Internet of things.

The memory capacity of the simulation experiment in this article is set to 5000, the maximum number of iterative steps per round is set to 1000, and the number of iterations is a total of 30 rounds. When the memory capacity reaches the maximum, the neural network starts to learn according to the data in the memory, and the new data obtained are covered on the basis of the original database, as shown in Figure 4. It can be seen from the results in the figure that when the number of iterations reaches the 16th round, the feedback reward value curve increases sharply until it reaches the maximum value and gradually tends to a stable state in the 19th round. This shows that the deep reinforcement learning computing migration algorithm based on the edge computing of multiple data sources in the Internet of things can find the scheme with the lowest energy consumption and the optimal scheduling of edge computing resources in a short time, which shows an ideal training effect and has a fast convergence speed. Resource allocation and task scheduling optimization are important research issues in computing systems. The scheduling problem attempts to allocate the maximum tasks in the edge computing structure. By deploying the deep learning layer in the edge server of the Internet of things, the transmission required by each task can be guaranteed.

**Figure 5:**
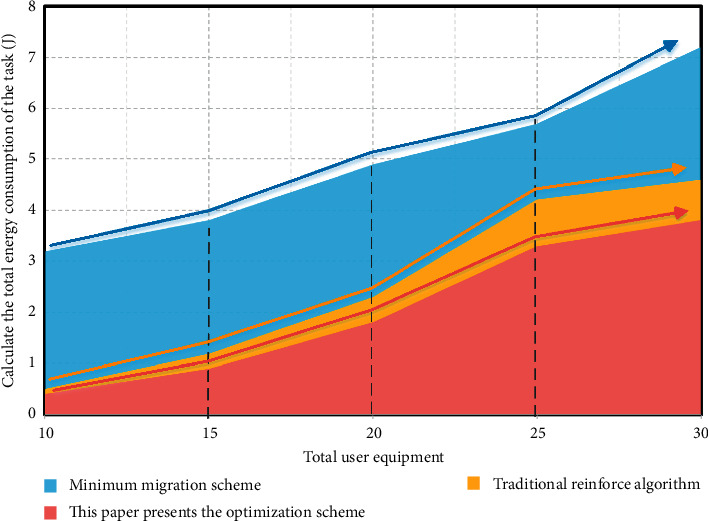
The relationship between the energy consumption of the three algorithms and the number of user devices to be migrated.

In order to better understand and study the performance of the deep reinforcement learning computing migration model based on the edge computing of multiple data sources in the Internet of things, this article compares it with the traditional reinforce algorithm and minimum migration scheme.  Figure 5 shows the relationship between the energy consumption of the three algorithms and the number of user devices to be migrated. It can be seen from the figure that the total energy consumption of either algorithm will increase with the increase of the total number of user devices. This is because the increase of the total number of user devices increases the amount of computing to be migrated, but the number and computing power of edge servers are limited. In this way, some calculations cannot be migrated and can only be performed locally. Compared with the other two migration schemes, the deep reinforcement learning computing migration scheme based on the edge computing of multiple data sources in the Internet of things consumes relatively less energy in terms of total energy consumption of resource scheduling, and the minimum migration scheme has the highest total energy consumption.

**Figure 6:**
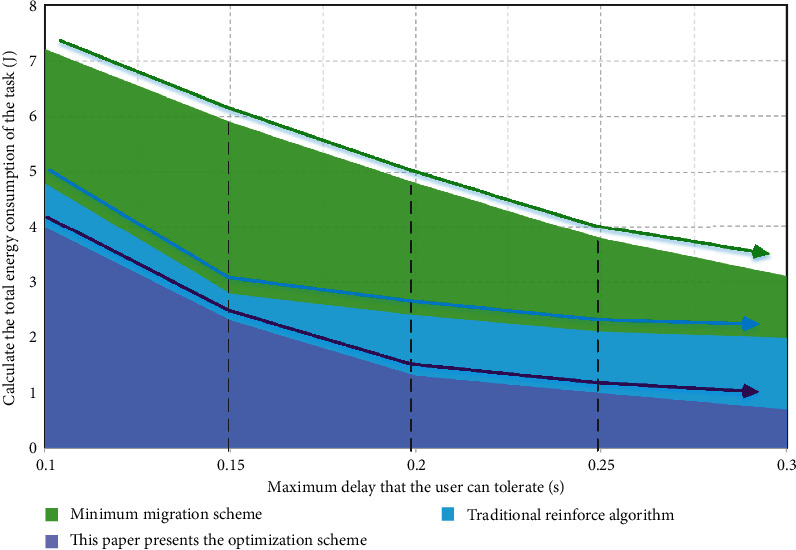
The relationship between the energy consumption of the three migration schemes and the maximum delay constraint that all users can tolerate.

Figure 6 shows the relationship between the energy consumption of the three migration schemes and the maximum delay constraint that all users can tolerate. It can be seen from the results in the figure that the total energy consumption of the computing tasks to be completed in the three computing migration optimization schemes will gradually decrease with the increase of the maximum delay constraint that all users can tolerate. This is mainly because some devices with poor channel conditions and limited local computing capacity can achieve maximum constraints. When the maximum delay constraint that users can tolerate increases, user equipment that is not suitable for migration computing can reduce the total energy consumption of the optimization scheme without calculating the impact of migration. Compared with the other two migration computing optimization schemes, the total energy consumption of the deep reinforcement learning computing migration optimization scheme based on the edge computing of multiple data sources in the Internet of things is the lowest.

**Figure 7:**
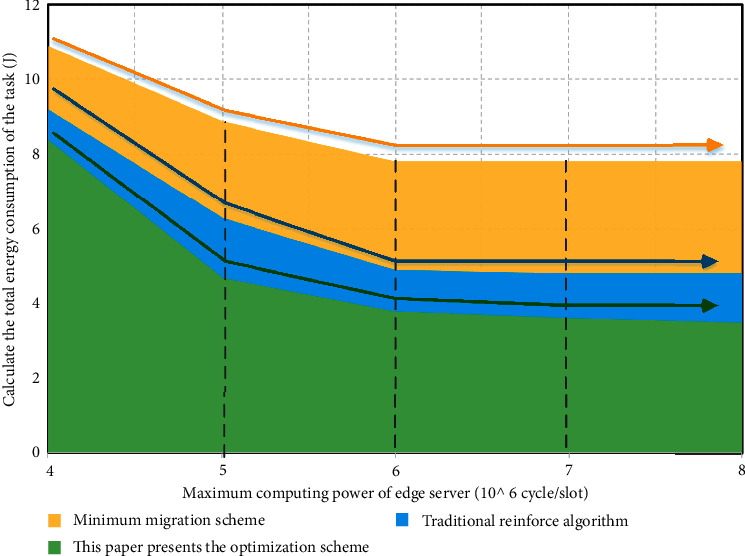
Relationship between energy consumption of three migration computing optimization schemes and the upper limit of computing power of edge servers.

Figure 7 shows the relationship between the energy consumption of the three migration computing optimization schemes and the upper limit of the computing power of the edge server. It can be seen from the figure that when the upper limit of the computing power of the edge server continues to increase, the total energy consumption required by the three migration computing optimization schemes to complete the corresponding computing tasks is gradually reduced. This is mainly because there are some user equipment with good channel conditions and suitable for migration computing. Because the edge server reaches the upper limit of computing, it has to choose the user equipment for local computing. When the upper limit of computing power of the edge server increases, the user equipment in this situation can choose migration computing, reducing the energy consumption required to complete the computing task, so as to reduce the total energy consumption of the scheme. Among the three migration computing optimization schemes, the deep reinforcement learning computing migration optimization scheme based on the edge computing of multiple data sources in the Internet of things requires the lowest total energy consumption for resource scheduling. It should be noted that the total energy consumption will not always decrease with the increase of the upper limit of the computing power of the edge server. When the upper limit of the computing power of the edge server reaches or exceeds a certain threshold, the total energy consumption of the optimization scheme tends to a stable state, almost reaching the situation that the consumption is no longer reduced. This shows that there is a stable state in the relationship between energy consumption and computing power of edge servers, that is, the upper limit of computing power of edge servers cannot be increased all the time but there is a critical value. When the upper limit of the computing power of the edge server exceeds the critical value, the total energy consumption of the migration computing optimization scheme will not continue to decrease.

**Figure 8:**
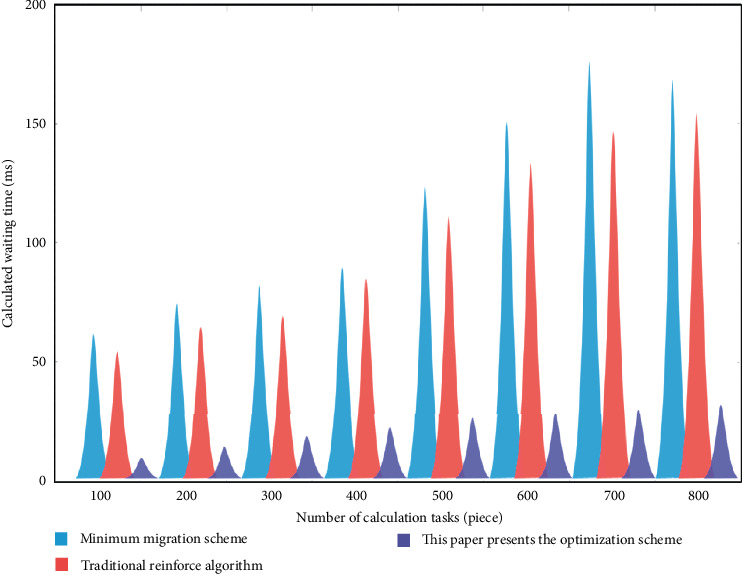
Comparison results of average calculation waiting time of three migration calculation optimization schemes.

Figure 8 shows the comparison results of the average calculation waiting time of the three migration calculation optimization schemes in the multiuser partial migration environment. It can be seen from the results in the figure that with the increase of the number of tasks, the average calculation waiting time of the three migration calculation optimization schemes shows an upward trend, and the overall increase of the other two migration optimization schemes is more obvious, However, the average computing waiting time of the deep reinforcement learning computing migration optimization scheme based on the edge computing of multiple data sources in the Internet of things increased slightly.

In the multiuser partial migration environment, when the number of tasks reaches 100, the average calculation waiting time of the traditional reinforce algorithm is 57 ms, the calculation waiting time of the minimum migration scheme is 65 ms, and the average calculation waiting time of the deep reinforcement learning calculation migration optimization scheme based on the edge calculation of multiple data sources of the Internet of things is 9 ms. When the number of tasks increases to 800, the average calculation waiting time of the traditional renew algorithm increases to 165 ms, the calculation waiting time of the minimum migration scheme increases to 180 ms, and the average calculation waiting time of the deep reinforcement learning calculation migration optimization scheme based on the edge calculation of multiple data sources of the Internet of things increases to 33 ms. The results show that the deep reinforcement learning computing migration optimization scheme based on the edge computing of Internet of things multiple data sources can optimize the ability of resource allocation and management based on the edge computing of Internet of things multiple data sources, improving the efficiency of edge computing server while maintaining uniform computing speed.

The relationship between comprehensive energy consumption and the number of user equipment to be migrated, the upper limit of computing power of edge server, and the maximum delay constraint that all users can tolerate can be obtained from the analysis of test results and calculated average waiting time test results, The deep reinforcement learning computing migration model based on the edge computing of multiple data sources in the Internet of things can effectively control the computing cost, reduce the overall energy consumption, and efficiently complete the required edge computing.

## 5. Conclusion

The development of 5G technology and Internet of things has promoted the implementation of the concepts of smart city and smart home, and the number of intelligent terminal devices is growing exponentially. When the local computing power and battery life of user devices are limited, the traditional cloud computing framework can no longer meet the needs of user intelligent devices for computing power. Edge computing migration and resource allocation management can not only provide users with better computing services but also reduce computing waiting time and energy consumption. Therefore, this article proposes the research on deep reinforcement learning computing migration and management based on edge computing of multiple data sources in the Internet of things by introducing the deep reinforcement learning technology into the edge computing algorithm based on multiple data sources of the Internet of things to optimize the algorithm, reduce the overall energy consumption, and improve the computing efficiency. The test results show that the overall energy consumption required by the deep reinforcement learning computing migration optimization scheme based on the edge computing of multiple data sources of the Internet of things increases with the increase of the number of user equipment and decreases with the increase of the upper limit of the computing power of the edge server and the maximum time delay constraint that all users can tolerate, Compared with the traditional reinforce algorithm and minimum migration scheme, the overall energy consumption is the lowest. In addition, the average computational waiting time of the algorithm increases slightly with the increase of the number of tasks. This shows that the deep reinforcement learning computing migration model based on the edge computing of multiple data sources in the Internet of things can effectively control the cost of edge migration computing and maintain a uniform and efficient operation while reducing energy consumption. However, the algorithm in this article still has some limitations. Deep reinforcement learning of data source edge computing the design of reward function of computing migration model is difficult. Reinforcement learning usually requires rewards, and the sampling efficiency of reinforcement learning is not clear enough, so it needs further discussion in future research.

## Data Availability

The data that support the findings of this study are available from the author upon request.
